# Patients With Rare Diseases and the Power of Online Support Groups: Implications for the Medical Community

**DOI:** 10.2196/41610

**Published:** 2023-09-14

**Authors:** Sadaf Ashtari, Adam Taylor

**Affiliations:** 1 Information Systems and Business Analytics department California State University, Sacramento Sacramento, CA United States

**Keywords:** application development, chronic pain, database, EDS, Ehlers-Danlos syndrome, genetic disorders, health care provider, information technology, online peer support group, privacy and security

## Abstract

**Background:**

Previous studies have shown positive tangible health benefits from using online support communities for informational support, daily living support, and emotional support. The specifics of how these communities can be improved have not been studied in detail.

**Objective:**

This study will investigate various sources of information that patients with genetic disorders use to learn more about their condition. We will be using patients with Ehlers-Danlos Syndrome (EDS) as a typical representation of the wider group of patients with genetic disorders. This study will also investigate the benefits and disadvantages of web-based platforms and how they can be improved.

**Methods:**

We used quantitative and qualitative analyses in this study. We undertook a web-based questionnaire survey and semistructured qualitative interviews through Zoom. Questionnaire results were analyzed using descriptive analysis. Thematic coding with constant comparison was used for interview transcript analysis.

**Results:**

A total of 436 respondents completed some or all of the survey. The majority of participants are female (386/413, 93.46%), and 24% (99/413) of them are in the age range of 25-34 years. Around 81% (336/413) of the participants have some type of college degree, and 55% (227/413) of them have graduate degrees. About 49.31% (204/413) of them are not currently employed. Most patients stated that their health care providers did not give accurate and complete information to them regarding their health situation (mean 2.87, SD 1.34). Also, patients perceived their providers as not knowledgeable regarding web-based communities that discuss patients’ conditions (mean 1.93, SD 1.15). Patients are confident in using health care resources available in web-based health communities (mean 3.78, SD 1.13). We interviewed 30 participants. The demographics of the interviewees were aligned with those of the survey participants. A total of 9 different themes were identified based on the Qualtrics survey and qualitative interviews. Participants shared the pros and cons of different online support groups that they were using and gave suggestions for improvement. They requested a centralized database with different categories of resources classified based on different diseases. They also emphasized the importance of search features and the ability to find relevant information with a hashtag. Furthermore, they elaborated on the privacy and security concerns they have regarding web-based support group platforms.

**Conclusions:**

Patients with rare diseases are finding information not available from their health care providers in community support groups. The medical community and web developers have a great opportunity to help these people by engaging with their web-based communities.

## Introduction

Rare diseases, about 72% of them being genetic disorders, impact 30 million Americans [1], bringing with them daily pain and discomfort that are not typically effectively addressed by the health care community [2]. Rare diseases pose significant challenges for patients, families, and clinicians in achieving accurate diagnoses and providing optimal care due to a lack of expertise and effective treatments [3,4]. Only 5% of rare diseases have a Food and Drug Administration-approved drug treatment [5]. Embodying the issues encountered by all patients with genetic diseases are patients with Ehlers-Danlos Syndrome (EDS), a genetic connective tissue disorder that creates structural defects in skin, joints, blood vessels, and other organs. Symptoms of EDS often include fatigue, weakness, damaged skin, joint dislocations, and chronic pain [6]. With no cures or significant treatments for genetic disorders, patients focus on mitigating pain and improving their quality of life [7]. Of great concern to patients with EDS in particular and those with rare diseases in general is the lack of disease information available to both themselves and their care providers [8].

The importance of online support groups has been studied among diverse groups [5] including diseases like Parkinson disease [7], psychiatric disabilities [9], Amyotrophic Lateral Sclerosis (ALS) [10], breast cancer [11], chronic diseases [12-14], neuromuscular disorders [15], and alcoholism [16]. Studies [11,12,17,18] found a positive impact of online support groups resulting from the emotional and community support found in these forums. Online support groups are also used for informational support. People with problems can share solutions to daily life challenges, suggest ways to cope with symptoms, and provide disease information to other members of the group [10,19].

In a previous study [20], the authors interviewed 30 patients with EDS and discussed their experiences with the health care system and how they coped with a lack of information about their disease. The vast majority found great use in online support groups hosted by Facebook and Reddit, as well as the Ehlers-Danlos Society web page. Access to information shared by other patients with EDS and sharing their own information had many positive health impacts.

Web-based information users were able to assist their health care providers in narrowing in on a diagnosis of EDS, the symptoms of which many had experienced for years either without diagnosis or with the wrong diagnosis. Patients were able to find and share academic articles and studies related to their condition, which allowed them to share the latest information with their caregivers. Referrals could be found for physician specialists as well as physical therapists with expertise in handling EDS symptoms. The availability of scientific data allowed patients to better collaborate with their health care providers, resulting in improved management of their disease [20].

Aside from managing the medical side of their disease, the previously mentioned study of patients with EDS [18] showed they were exposed to a plethora of information that helped improve daily living. Online peer support groups were excellent sources of solutions to the daily pain, discomfort, and overall inability to perform daily life functions [8]. Most patients had tried traditional pain medication and, finding this to be insufficient in relieving pain and presenting them with undesirable side effects, discovered things like acupuncture, cannabis, targeted exercise, physical therapy, massage, and diet as an effective supplement to or replacement for traditional pain medication. The informational sites gave recommendations for driving, positions for sleeping, and support for mobility devices that had a significant impact on the ease with which patients with EDS could perform everyday tasks [20].

Online support groups also provide a forum for emotional support. These sites were a place where people with similar experiences could gather in a community. Most of the interviewees were not as interested in hearing negative stories as they were in finding solutions. Support groups were a place to find positive, like-minded individuals. The community gave the participants a safe location to express their feelings and receive feedback. The sharing of information resulted in a sense of satisfaction and the exertion of control over negative circumstances, increasing the positive emotional outlook [20].

Information sharing and web-based engagement is demonstrated to be beneficial to the health and well-being of patients with EDS. This study investigated the facets of online support groups that were of concern to patients with EDS and the changes that could be made to increase the likelihood of sharing. The issues of security, credibility, organization, searchability, and usability were the highest priorities. This study recognizes 2 groups that could have the greatest impact to increase sharing on support groups: website developers and medical professionals. Platform developers would improve information sharing by creating support group websites where information was highly categorized and easily searchable. Concerns about the security of users’ identities and health information could also be addressed with appropriate security measures implemented by application designers. Medical professionals, by participating in generating and moderating web-based content, would lend a high degree of reliability to the information presented on these sites. Patients would also benefit from being directed to high-reliability sites by members of the medical community.

Though we only examined patients with EDS as a sample of the overall experiences of patients with genetic diseases, the intent of future studies will be to examine other genetic disorders to see if their experiences and the benefits of online support groups are comparable.

## Methods

### Overview

We conducted a cross-sectional qualitative and quantitative study using an electronic survey that was designed for the purpose of this study. Descriptive analysis has been used to evaluate survey data. For the qualitative part, we performed individual, semistructured interviews with patients with EDS to understand the different sources of information they were using and the pros and cons of each. Transcripts were analyzed using a thematic analysis approach.

### Participants and Setting

We sent the study flyer to the Ehlers-Danlos Society website. The website’s administrators shared the recruitment announcement on their main website and other affiliated web-based platforms. Participants were invited to participate in the study if they were 18 years of age or older, had a diagnosis of any type of EDS, and are members of any online support group.

Participants volunteered for the study by clicking the survey link in the recruitment flyer and were directed to a web-based questionnaire via Qualtrics. The survey lasted from March 2020 until April 2021. In the study announcement letter, interview volunteers were instructed to email the primary investigator to express their interest and learn more about the study. Further communications took place to set up a 40-minute interview through Zoom. From July 2021 until December 2021, out of 57 emails received, a total of 30 appointments were scheduled. The rest of the initial email responders never replied with their availability.

Based on a previous study [18], we focused on patients and not health care providers. The overarching theme of the previous study was that providers were generally well-educated in rare diseases and provided minimal direction in pain management other than prescribing opioids. The goal of this study is to discover the ways patients use online support groups to supplement their existing medical care.

#### Quantitative Data Collection

We developed the structured questionnaire through Qualtrics, which consisted of 2 major parts. The first section, demographic information, contained 7 questions. The second section consisted of 26 questions related to the different sources of information patients are using, their participation level, engagement, and pros and cons of the web-based platforms. The questions for the second section are listed in Table 1, sorted by issues in the same order presented in the questionnaire.

**Table 1:** Items of the second section sorted by issues.

Issues	Items	Measurement
Sources of information	Where do you usually find help about your disease or condition? (Name online or offline sources of information.)	Open-text field
Online peer support group usage	Are you part of any online peer support groups? (Yes, no) name it. If you answer is yes, answer the following questions.	Open-text field
Information from health care providers	My health care provider gives me accurate and complete information regarding my health situation. My health care provider shares meaningful information with me. My health care provider is knowledgeable regarding online communities related to my conditions.	5-point Likert scale
Self-reliance in accessing information	I know to whom I can turn to when I have a health problem. I know how to use the health resources available to me in the online health community. I know how to access resources such as information, money, services, or support for dealing with health problems.	5-point Likert scale
Participation and engagement in online peer support group	I feel I am part of the online Ehlers-Danlos syndrome support group. I am proud to tell people I am on the online Ehlers-Danlos syndrome support group. I would be sorry if the online Ehlers-Danlos syndrome support site shut down.	5-point Likert scale
Willingness to share information	I feel satisfied when I share some knowledge about Ehlers-Danlos syndrome with other members in online support group. I would share my personal experiences about online support group with other members. The interesting information about Ehlers-Danlos syndrome can motivate me to share it.	5-point Likert scale
Ease of use	Online Ehlers-Danlos syndrome support group is convenient. Online Ehlers-Danlos syndrome support group makes it easy to communicate. Online Ehlers-Danlos syndrome support group allows me to convey emotion. Online Ehlers-Danlos syndrome support group allows me to connect with multiple other members.	5-point Likert scale
Information use	I am likely to use the information obtained from the online Ehlers- Danlos syndrome support group. I am satisfied with the information obtained from the online Ehlers- Danlos syndrome support group. Online Ehlers-Danlos syndrome support group allows me to control how much information I receive from others. Online Ehlers-Danlos syndrome support group allows me to filter out information if I want to.	5-point Likert scale
Privacy and security concerns	Online Ehlers-Danlos syndrome support group allows people to remain anonymous or unidentifiable if they want to. I am afraid if my friends or family read my post on online Ehlers-Danlos syndrome support group. I am anxious about losing my medical data when using the online Ehlers-Danlos syndrome support group. The online Ehlers-Danlos syndrome support group has a sufficient level of security.	5-point Likert scale

Patients provided their source of information and whether it was a web-based source or not. They provided the names of the web-based peer support platforms in which they were participating. Part 1 of the second section addressed the knowledge of health care providers regarding sharing disease***-***related information. After that, patients were questioned about their knowledge in accessing information. Their answers were rated on a 5***-***point Likert scale from 1 (“strongly disagree”) to 5 (“strongly agree”). Additional questions were asked if patients selected web-based sources of information. The rest of the questions asked about the patients’ participation, engagement, information use, sharing of information, and pros and cons of these platforms. We used a 5***-***point Likert scale for this section of the questionnaire.

Participants gave consent by clicking on the “continue” button when they entered the Qualtrics survey. It took individuals 15***-***20 minutes to fill out the questionnaire. The participants were offered the chance to enter to win one of ten US $30 Amazon e***-***gift vouchers in appreciation of their time.

#### Qualitative Data Collection

Data were obtained from semiinstructed interviews conducted by the first author of the study (SA) through Zoom conference call. We used open-ended questioning and determined the order of questioning by the direction taken by each interview participant. The question guide is presented in Multimedia Appendix 1. The interviews focused on (1) diverse sources of information regarding patients’ diseases and conditions; (2) health care provider roles in sharing disease-related information; (3) opinions about web-based peer support; and (4) the pros and cons of each platform.

Each interview began with sharing the experiences of the researcher (SA) as a genetic disorder patient with chronic pain and later moved to explore patients’ disease-related information needs and their challenges with pain mitigation and care management. The interview, including participant demographics, was recorded with the consent of participants. Each interview took around 45 minutes.

Survey and interview data were held in password-protected files on the primary investigator’s password-protected computer.

### Data Analysis

The survey results were analyzed to provide a summary of descriptive statistics. Means and SDs for continuous data and percentages for categorical data were generated.

All interview transcripts were read and coded independently by 2 investigators (SA and AT), who familiarized themselves with the data through reading and reflection. Constant comparison was used to refine emerging thematic frameworks. The investigators agreed on a series of thematic codes that described a number of categories and subcategories. The final codes were reapplied to the transcripts using NVivo 12 (software 12.2.0).

### Ethics Approval

All research activities were approved by the Sacramento State Institutional Review Board FWA00003873 (IRB protocol number: IRB-19-20-173).

## Results

### Overview

Following the quantitative and qualitative analysis, a total of 9 different categories were identified, such as (1) patients’ sources of information, (2) lack of attention from medical community, (3) self-reliance in accessing information, (4) participation and engagement in online peer support groups, (5) patients’ willingness to share information through web-based platforms, (6) platform improvement suggestions, (7) information use and validity or reliability, (8) necessity of centralized and categorized sources of information, and (9) privacy and security concerns.

### Questionnaire Survey

A total of 413 participants completed at least part of the survey. We removed the responses (23 surveys) that had a lower than 5% completion rate from the result. A total of 95 surveys had a completion rate of 33%, and 318 of them were completed fully. The incomplete parts of the survey have been considered as missing data in different analyses. Results are presented as a proportion of the total number of responses to each question; therefore, the denominator varies according to the individual question response rate. Participants characteristics are demonstrated in Table 2. Around 24% (99/413) of the participants are in the age range of 25-34 years. Of the participants, about 93.46% (386/413) of them are female and 89.59% (370/413) are of White ethnicity. While the sample population may not accurately represent the broader population of the United States or other countries, research indicates that female individuals with EDS are more likely to seek medical care than males, who experience less pain and fewer significant joint complications. The reason for this gender difference may be linked to differences in pain sensitivity, inherent joint stability, and the effects of sex hormones, as suggested in the literature [21]. EDS may be underdiagnosed in women due to medical bias [22,23]. In a 2009 survey of 414 families in 5 countries, women waited on average 16 years for a diagnosis, compared to 4 years for men. Women's physical symptoms are often overlooked and categorized as psychological or common complaints [24]. Although 81.35% (336/413) of the participants have some college degree and 55% (227/413) of them have graduate degrees, about 49.39% (204/413) of them are not currently employed. In the interview most of them revealed that chronic pain and health conditions prevented them from working full-time.

**Table 2:** Participants characteristics (N=413).

Participant characteristics				Participants, n (%)
**Gender**				
		Female		386 (93.46)
		Male		18 (4.36)
		Other		9 (2.18)
**Age (years)**				
		Younger than 18		19 (4.6)
		18-24		80 (19.37)
		25-34		99 (23.97)
		35-44		95 (23)
		45-54		71 (17.19)
		55 and older		49 (11.86)
**Ethnicity or race**				
		Asian		1 (0.24)
		African American or Black		4 (0.97)
		Latino		13 (3.15)
		White		370 (89.59)
		Other		25 (6.05)
**Highest level of education**				
		Less than high school		13 (3.15)
		High school graduate (included equivalency)		34 (8.23)
		Some college, no degree		105 (25.42)
		Associate’s degree		30 (7.26)
		Bachelor’s degree		122 (29.54)
		PhD		18 (4.36)
		Graduate or professional degree		91 (22.03)
**Employment**				
		35 hours a week or more		128 (30.99)
		Less than 35 hours a week		81 (19.61)
		Not currently employed		204 (49.39)
**Country of residence**				
		International		106 (25.67)
		**United States**		307 (74.33)
			Midwest	79 (25.82)
			Northeast	77 (25.16)
			Southeast	60 (19.61)
			Southwest	26 (8.50)
			West	64 (20.92)

### Interview Sample

The descriptive characteristics of our interview sample were aligned with the quantitative survey. The majority of interviewees were identified as female (28/30, 96%). Twenty-eight (28/30, 92%) participants described their ethnicity as White, and 2 belonged to other ethnic groups. About 77% (23/30) of participants reside in the United States. Participants ages ranged from 18 to 69 years old. Similar to the quantitative result, more than 66% (20/30) of participants have some college or graduate degree, but less than 44% (13/30) of them are currently working.

### Different Sources of Information

Based on the patients’ responses in interviews and surveys, we sort the sources of information that patients are using to learn about their disease and educate themselves about their condition into 2 categories: web-based and offline sources. The main offline sources are patients’ medical teams, such as primary care physicians, physical therapists, massage therapists, and chiropractors. Patients talking to peers and reading books are another example of offline information resources for patients with EDS. The web-based sources are classified into 4 groups: (1) journals and PubMed publications; (2) organizational websites (EDS Society, National Institute of Health [NIH], WebMD, and Inspire); (3) online peer support groups hosted by different platforms (Facebook, Twitter, Reddit, and Instagram); and (4) Google search. More than 50% (206/413) of participants mentioned that their main source of information is the EDS Society website. A total of 360 (88.67%) survey respondents declared that they are members of 1 or 2 online peer support groups. A total of 330 (59.78%) participants are members of the Facebook EDS support group. Participation in other support groups is as follows: Inspire (n=75, 13.59%), Reddit (n=42, 7.61%), and Patients Like Me (n=6, 1.09%). Respondents shared other support groups, but the number of participants was less than 1%, so we did not report it. Some of the names are Instagram, Chronic Warrior Collective, EDS Connections, EDS Zebras need Zebras, The Mighty, TikTok, My Fibro Team, and Rare Connect.

### Lack of Attention From Medical Community

The survey responses to the second section, question 2-3, revealed that health care providers did not give accurate and complete information to patients regarding their health situation (item a: mean 2.87, SD 1.34). In addition, health care providers didn’t share meaningful information with them (item b: mean 2.35, SD 1.33). Providers are not knowledgeable regarding web-based communities that discuss patients’ conditions (item c: mean 1.93, SD 1.15). Of the 413 patients recruited, 394 responded to these questions, and our descriptive statistics showed that most respondents perceive their health care providers’ knowledge of the disease and their effort to share relevant information negatively (Multimedia Appendix 2, Table S1).

During the interviews, participants shared different experiences with their medical teams. The majority of them expressed dissatisfaction with their health care provider. One of the interviewees depicted her experience as follows (All quotes are presented verbatim, but to improve readability, some filler words such as like and um have been removed. Also, the information in the brackets at the end of each quote refers to the interviewee’s information that we de-identified and coded):

I went from doctor to doctor to doctor, none of my doctors know much. My specialist couldn’t barely pronounce any of my issues. I have my primary care physician who's just a regular doctor, but she knows very little and has done almost nothing.

SA-MA

Most of the patients were frustrated and overwhelmed by not receiving the required information from their health care professionals.

In Australia EDS isn't particularly well known by doctors, you have to see a rheumatologist and last time I had to see him, it was six months on the waiting list.

SA-AU

It is surprising for patients that, despite the large number of people with genetic disorders around the world, there is little support from the medical community.

It feels there is no support from the medical community and that people are just kind of grassroots it just feels like.

AM-CO

I am surprised by the sheer numbers of people because I always understood that it was quite a rare condition. But even for the Netherlands, which is quite a small country, the group is huge, hundreds of People on there.

NY-NE

I am amazed by the lack of medical professional support that we all have and feeling that a lot of us are being told it's all in your head you're depressed, and you have anxiety. So, my fear is that something could majorly be wrong with me health wise, and I don't go in, because I’m afraid to deal with that and I could have something seriously wrong with me.

PA-MI

### Self-Reliance in Accessing Information

Respondents to the Qualtrics survey declared they know who they can turn to when they have health problems (item a: mean 3.50, SD 1.24). They are confident in using health care resources available in web-based health communities (item b: mean 3.78, SD 1.13) and are confident in accessing information (item c: mean 3.11, SD 1.29). Of the 394 respondents, more than 60% of them agree or strongly agree they know how to access and use information related to their disease and care management (Table S2 in Multimedia Appendix 2).

In the interviews, patients mentioned that whenever they face a health issue, they find information on Google and Google Scholar and then share their findings with their doctors. Most of the time, they are not receiving useful feedback.

I’m really good at searching, so I know that I can go to Google and type in Ehlers Danlos society webinar. Whatever topic I’m looking for and then that way Google will literally give me the results right whether it's EDS society on YouTube or on their webpage exactly and so that's one way that I do it.

SA-CA2

I'm just going through Google scholar and pulling up various different studies and stuff but also looking at some of them are older and seeing if they've come out with more recent.

EL-MA

The majority of it is doing research online or on the Ehlers Danlos society website.

PA-MI

One of the patients told the author (SA): “There are not many reliable and useful sources and that’s why you are doing this research.” Most of the patients emphasized the importance of this study as a great opportunity for them to talk about their problems and find more optimal solutions.

### Participation and Engagement in Online Peer Support Groups

Patients expressed their interest in participating with and engaging in online support groups and communities. They conveyed a sense of belonging to these communities. They feel they are part of the online EDS support groups (item a: mean 3.23, SD 1.24) and are proud to tell people that they are members of these groups (item b: mean 3.51, SD 1.19). Of the 382 respondents, about 300 mentioned they want these types of communities to continue and would feel sorry if they shut down (item c: mean 4.41, SD 1.09). The details of descriptive statistics are shown in Table S3 in Multimedia Appendix 2.

### Willingness to Share Information

Most of the participants in the survey and interview agreed that having a community, giving and receiving support, and sharing their experiences with each other was one of the main reasons they were part of these groups. A total of 74% (231/327) of participants feel satisfied when they share some knowledge about EDS with their peers (item a: mean 4.0, SD 0.93). About 65% (212/327) of respondents declared that they would share their personal experiences about online support groups with other members (item b: mean 3.72, SD 1.09). Also, the majority of them (255/327, 78%) stated that interesting information about EDS can motivate them to share it (item c: mean 4.07, SD 0.9). Further descriptive data are demonstrated in Table S4 in Multimedia Appendix 2.

During the interview, participants elaborated on their willingness to share information. Although sharing experiences and having a community is an important aspect of contributing to these communities, some of the participants felt uncomfortable, having some privacy concerns about sharing their personal information. We will discuss the privacy and security issues in more detail later in this study.

Almost half of the interviewees found helping people and sharing information with them rewarding and satisfying.

I give other people advice when I see people posting and asking because that's what they'll do like hey guys, I have this this this going on, you know? I am able to help people because I see these people that are just starting on their journey or there are much younger than me and they've been diagnosed, so I can give them these tips that are all my best practices that I’m finding out through all these experts. so, it also gave me a sense of purpose.

SA-CA

I really, really felt good about helping people. it's very, very gratifying. Let's share information. Let's share resources. You've been to this doctor, what did you learn it offers all of those opportunities to help.

MI-MA

I feel like I need to help people.

DA-IT

A sense of community and a feeling of belonging are important for members of online peer support groups. It helps with their mental health.

It really helps to hear, even if it's just from one other person that you're not alone, this isn't pretend, it's not in your head, and will help you. It's a big deal.

SA-CA2

You know people sharing, you know what's bothering them, and other people with input by large, you know being very respectful of differences. I really felt at times I inhabited another world, when the pain is so great and it just colors everything, I felt very detached even from my husband.

SU-NY

I was like I’m normal within my subset of people. I’m the majority here, and so that was kind of cracking me up because it's like you've been the outcast. And so, it actually helps with the mental health aspects. On Instagram just knowing that your part of this huge Community there are hundreds of thousands of other people out there going through the same thing as you.

SA-CA

Sharing personal experiences is a huge benefit for many patients who are going through the same situation.

It just kind of reading people's accounts of what they've experienced and sort of trying to figure out how that relates to me it does.

AM-CO

Some days, sometimes people share their stories. It can be my stories well it's the same, also your diagnosis is almost similar. Our stories are happening in different kinds of places in the world, but they're very similar.

DA-IT

I like when people have recommendations or what works for them. lot of times we'll have the same injuries or the same issues, so they usually have a wide variety of ways to deal with it, that most doctors wouldn't think of.

JE-AL

I want to know what that's like for other EDSers. I want to know how it lasts for them, I want to be able to see their experiences and find what they write about it more easily. To be honest, sometimes, I just wish they were older people that had EDS so I could see what my life might be like in 15 or 20 years because.

MI-SE

### Platform Improvement Features

The current online support group platforms have some features that can encourage people to participate, share information, and give and receive support. However, there are still many ways that platforms developers, with the help of health care professionals, can improve these applications. Around 86% (290/336) of survey participants thought that online EDS support groups are convenient (item a: mean 4.24, SD 0.80) and make it easy to communicate (item b: mean 4.05, SD 0.9). More than half of the respondents (213/336, 63%) believed their online peer support allowed them to convey emotions (item c: mean 3.74, SD 0.98) and 84% (282/336) found it easy to connect to other members through their platform (item d: mean 4.25, SD 0.82). The details of descriptive statistics are revealed in Table S5 in Multimedia Appendix 2.

All of the patients in the interview mentioned that they feel comfortable working with these applications. They like the threaded conversation feature and also use emojis. Writing comments, sharing a post, and direct messaging are other features of these platforms which patients found useful. However, they think there are many features that can be added to the current platforms, or a new platform can be created that uses these features. One of the main features people like to use to find relevant information is a hashtag. Right now, information is not classified based on category. Having this feature and educating people on how to use it makes information access faster and easier.

Most people don't think to tag their comment or their story, or they might not know how and so automatic tagging work. I think would help to make it more searchable. Because truthfully the same new people get on every day and they're asking maybe the same questions and if you've been in a support group a long time you could get tired of that.

LY-NA

Keep recycling the good information that's on there. Because it does become relevant over and over and over again. And, if somebody has an experience, maybe they don't remember the post from three weeks ago, or three years ago that really answered their question until they personally have to deal with that issue.

MI-MA

Another feature patients like to have, especially after the COVID-19 pandemic, is the option to have a Zoom call with their peer. Feelings of isolation are inevitable when they only communicate through text messages and comments.

I found that's been helpful to have zoom meeting. You are actually meeting face to face with people that have Ehlers Danlos or maybe we'll be able to talk about things that are going on.

SA-TE

Since we've been working from home. I've had to get used to Zoom call and it’ll be helpful to talk to somebody face to face and having a conversation.

AY-MA

Other features patients think would be helpful are the ability to pin the most liked posts, the availability of a more advanced chat box (a combination of voice, text, and video), and having a doctor and medical staff tab. Several patients suggested that having medical professionals involved in these platforms would be very helpful in terms of offering advice and sharing knowledge.

The useful part would be the files and the recommendations from medical team that you can search for within the group. Asking directly if you know who I can go see for this and getting the different recommendation.

PA-MI

I wish there were medical people that were involved, like doctors that are involved in each one of the groups that could give recommendation. I know that they can't offer advice online but maybe give direction, hey go here, call here, you know not saying this is what you probably have but call this type of a doctor, I think that would be really helpful.

SA-SC

### Information Use and Reliability

As we discussed earlier in this study, the medical community’s lack of knowledge about their genetic disorders forced patients with rare conditions to obtain information by themselves, often through web-based resources. Participants (311/364, 85.44%) are positive about the usage of information which they obtain through online peer support groups (item a: mean 4.22, SD 0.82). The majority of them (266/364, 73.08%) are satisfied with the information obtained from these platforms (item b: mean 3.90, SD 0.92). The opinion of survey respondents regarding filtering and controlling the amount of information received is not completely aligned with the interviewees’ responses. Qualtrics results showed that 48.81% (164/336) of respondents believed current platforms allowed them to control the amount of information they were receiving (item c: mean 3.37, SD 1.04). Also, 42.26% (142/336) of participants agreed that the current online support group platform allows them to filter out information if they want to (item d: mean 3.17, SD 1.11). See Table S6 in Multimedia Appendix 2.

Interview respondents have some concerns about filtering and how that results in wasting time trying to find the right information.

I really try to filter and ignore a lot of posts that I don't really think make sense to me. It's not super straightforward. I don't have a difficult time with it, although I find it time consuming, because I do have to skim a lot of posts before I find something that I think is useful.

AN-AP

I'm going to say the filtering system that they use to filter the posts, it just

isn't great. There are a lot of repetitive posts. There are a lot of posts that get past that shouldn't. And there are a lot of good posts that get stuck in the filter.

SA-MA

Patients focused on the importance of search features and the ability to find relevant information for their cases with a hashtag. They are also looking for up-to-date information that they can rely on.

It would be nice to have an updated directory. I know a lot of websites try to put specialist name, but you have to keep that updated and all that stuff, but we just don't have that in Canada.

JE-AL

The validity and reliability of information are very important for users of online support group platforms. They want to have a source of information that is validated by research or health care professionals.

I think it would be really helpful for me if some of the treatment was validated by research, because a lot of these people are coming up and saying this helps me but there's no validity to the treatment and I don't trust that, and I find that more frustrating so when I go to ensure that the solutions are valid and not just somebody saying oh I eat berries and healthy.

AM-CO

Online resources can be useful and useless, at the same time, you have to know, when you search Google, you have to read and analyze and then decide if it's useful or not, because sometimes they spread misinformation too.

DA-IT

I'm a huge skeptic, especially if you're going to bring me information that I've dealt with for a good number of years of my life.

AY-MA

### Necessity of Centralized and Categorized Source of Information

There is no doubt that patients are receiving considerable benefit from web-based resources; however, given their important role in the lives of patients with rare diseases, it is essential to improve the current platforms or build new ones with the requested features. Out of all the features patients requested, having a centralized database with different categories of resources, classified based on different diseases, is the most important. They stated they have problems finding or searching for reliable information, but all agreed having a single, up-to-date database with proper categories is the most useful.

It would be great if there was a central resource, where I could go and say, these are my symptoms, this is what I’ve been diagnosed with and what works for these people, because the issue is that we're all kind of being grouped into one. For chronic patients who are in extreme pain, there needs to be a centralized resource to drill down based on your specific issues, because eds is a spectrum disorder.

AM-CO

It would be nice to have everything condensed in one place and you're not missing anything if you just kind of want to scroll through. I don't want to spend that long on Facebook. I don't want to have to click every group individually and scroll through and see what I've missed. I wanted to be able to kind of go through and just pick through.

EL-MA

Because the needs of patients are different and they are looking for different types of support (informational or emotional), it would be helpful to classify the information based on purpose.

That's all I can come up with, I wish that they would organize the post by what they mean. What they have what they asked for.

SA-MA

I would prefer a platform that incorporates through, but at the same time keeps them separated. That's probably because now, I have to choose one or the other, and it would be great if you had a platform that incorporated both elements.

NY-NE

They need to section off so if you need emotional support go over here. if you need practical support, go over, if you need a recommendation go over here if you need medicine go over there.

JU-KE

One of the patients described her challenges:

I should be able to find stuff easier. But that's the thing, I have to go to the Facebook and then go to the Ehlers Danlos Washington site and then click through to the Google files to find one file and then I go to the Ehlers Danlos society website and click. I don't have time for that. It can just be one place.

MI-SE

### Privacy and Security Concerns

Privacy and security concerns make some people uncomfortable about sharing their personal information. Others have no issue with it. Different platforms have different policies for creating accounts for their users. Some may allow users to have a fake name and an anonymous avatar, while others do not. As with most everyone, not every user takes time to read the site policies, so respondents were neutral about whether they could remain anonymous or unidentifiable (item a: mean 3.01, SD 1.28). More than half of the survey respondents (172/318, 54%) are not afraid of their friends or family reading their posts in online peer support group (item b: mean 2.45, SD 1.30). The majority of them (207/318, 65.09%) also stated they are not anxious about losing their medical data when using the EDS support groups (item c: mean 2.08, SD 1.11). Most of our participants were neutral about the security level of EDS online support groups (item d: mean 3.08, SD 0.90). See Table S7 in Multimedia Appendix 2.

Interviewees had a chance to elaborate more on privacy and security concerns. One of their main concerns is the existence of fake accounts. They do not feel comfortable trusting people they have never met in the real world.

I got scared because I didn't want somebody to post me on reddit and say that I’m a fake person and I don't want to post my private medical records to prove myself so.

HE-CA

Another concern that they raised is about sharing their identifiable information on the internet. They prefer to share less information about themselves that could be used to identify them.

Being able to use a nickname and having an Avatar is fine, but not having it mandatory that I have to have my face, there are some support groups where you must have your profile picture as your face and stuff and I don't have that on social media. I don't necessarily put a lot of personal information out there, I certainly don't have a lot of identifying information.

LO-UK

It's kind of weird to have any personal information out there with a bunch of strangers but knowing it's a private group makes it a little bit better and only some of the groups that I'm a part of a private groups. But I had posted a screenshot of my shower stool that I ordered on Amazon and then I realized at the bottom it had my fiancé’s name and our zip code so I deleted it and post it again. It's not that anybody would have done anything but that kind of makes me uncomfortable having that information out there.

ER-MA

They also mentioned the difficulty in tracking privacy policy changes of the platforms they use.

I always worry a little bit about privacy. I definitely prefer apps that seem more secure. I'm not really sure I haven't stayed as up to date on the changes Facebook has made to their privacy policies. The inspire platform does seem to take privacy pretty seriously so that has made me more confident, but I also haven't looked into their updates more recently.

AN-AP

## Discussion

### Principal Findings

Based on a previous study by the authors [20], this study assumes positive mental and physical health outcomes as a result of participating with and engaging in online support groups. The aim of this study is to discuss the ways medical providers and platform developers can increase the engagement of patients with rare diseases, thereby improving outcomes.

Interviewees expressed concern about the quality and reliability of the information they were seeing, as well as having it presented in an intuitive way that was easy to search.

The top functionality the interviewees wanted to see was categorization and segmentation of content. Rather than manually searching discussion threads or lists of comments, users wanted to see content divided into related groups such as academic research, life skills, and provider referrals. To organize this further and assist in searching, many of the interviewees mentioned the use of hashtags for information sharers to further categorize their posts. Respondents expressed support for robust search capabilities to assist in finding the exact information of interest to them.

Patients with EDS expressed significant concerns about privacy on the internet. Survey respondents and interviewees wanted to make sure their identities could not be stolen and that they could remain anonymous if they wanted to do so.

Engagement by patients is significantly bolstered by engagement by health care providers. Our previous study [20] found that providers did not direct their patients to online support groups, did not generate content for those forums, and did not assist in moderating the content. Patients with EDS want the information in the support groups to be reliable and based on facts. They would feel confident if they knew answers and information were coming from a trusted source. They want misleading or false information to be flagged.

Of note are the economic costs of rare diseases. Most of the respondents and interviewees had college degrees, but half were not working due to their health conditions.

### Implications and Recommendations

Technical concerns about usability, function, and security are all addressable by improved design. The features desired by patients with rare diseases exist today in some form in existing support groups and other websites. These features have just not been combined into a single location specific to addressing patients with rare diseases’ needs. For instance, many websites allow users to select their privacy level, choosing between completely anonymous, partially anonymous, or completely open. Control over anonymity has been shown to increase the likelihood of information sharing [25,26].

Currently, health care providers are ambivalent about online support groups [27]. To improve patient engagement, the medical community needs to become involved in online support groups. They can refer new patients to these groups, answer questions, moderate existing content, and generate new content. The more patients with rare diseases trust the information they are given, the more of their own information they will provide, thereby increasing the overall amount of useful information.

Combining the 2 groups, platform developers can assist health care provider engagement by creating tools that make it easier for providers to add, review, and delete content.

Future studies will be performed on different genetic diseases to validate the findings of this study.

Future studies may also investigate the ability to harvest useful clinical data from online support groups for use in clinical disease research and treatment.

#### Questions for Future Investigations

Since the vast majority of participants were female, additional study should be given to the reasons for this discrepancy. The likely reason for this is not due to different EDS rates between men and women but to how the study was conducted. Advertisements for this study were posted on the EDS Society web page and affiliated social media platforms. A literature review of gender differences in online health support groups suggests men are typically more interested in medical information and treatment issues, while women are more interested in emotional support [28]. The perception of “support groups” as primarily for emotional support may be a factor. Future studies where participants are self-selected will need to examine advertisement location in relation to medical or treatment information as compared to emotional support content. Though demographic percentages did not precisely match the overall demographics of patients with EDS, our goal was to find heavy users of online support groups and document the benefits these patients received from participation. We would like to expand the demographic variety and explore different diseases in future studies.

### Limitations

The interview subjects represented a small sample size of 30. All participants in the interview and surveys were self-selected through advertisements on existing social media sites. All interviewees and 93.46% (386/413) of the survey respondents were female. The study only included patients with EDS as a representation of rare diseases.

### Conclusions

Engagement in high-quality, moderated online support groups is beneficial to the mental and physical health of patients with genetic disorders. Patients are already very engaged and innovative and are waiting for platform developers and health care providers to catch up to them. By improving usability, privacy, and reliability, patient engagement will increase, improving health outcomes and reducing social costs.

## Appendix

Multimedia Appendix 1 Interview question guide.

Multimedia Appendix 2 Descriptive statistics analysis.

## Abbreviations

ALS: Amyotrophic Lateral Sclerosis
EDS: Ehlers-Danlos Syndrome
NIH: National Institute of Health
