# Supplementary material for: Patients With Rare Diseases and the Power of Online Support Groups: Implications for the Medical Community
Source: JMIR Form Res. 2023 Sep 14;7:e41610. doi: 10.2196/41610 (PMC10540027; doi:10.2196/41610)
Supplement: Multimedia Appendix 2 [file formative_v7i1e41610_app2.docx]

Table S1. Descriptive statistics of survey 2-3 questions and responses.

| **2-3 Information from healthcare providers, (%), (n)** | | | | | |
| --- | --- | --- | --- | --- | --- |
| **Items** |  | | | | |
|  | Strongly disagree (1) | Disagree (2) | Neither agree nor disagree (3) | Agree (4) | Strongly agree (5) |
| Item a) | 19.54%  (77) | 26.49% (104) | 13.71%  (54) | 27.92%  (110) | 12.44%  (49) |
| Item b) | 36.29%  (143) | 25.38% (100) | 13.71%  (54) | 16.50%  (65) | 8.12%  (32) |
| Item c) | 49.75%  (196) | 22.84% (90) | 15.99%  (63) | 27.92%  (110) | 4.06%  (16) |

Table S2. Descriptive statistics of survey 2-4 questions and responses.

| **2-4 Self-reliance in accessing information, (%), (n)** | | | | | |
| --- | --- | --- | --- | --- | --- |
| **Items** |  | | | | |
|  | Strongly disagree (1) | Disagree (2) | Neither agree nor disagree (3) | Agree (4) | Strongly agree (5) |
| Item a) | 9.90%  (39) | 15.23% (60) | 8.63%  (34) | 47.21%  (186) | 19.04%  (75) |
| Item b) | 4.82%  (19) | 11.93% (47) | 13.20%  (52) | 40.86%  (161) | 29.19%  (115) |
| Item c) | 13.71%  (54) | 24.11% (95) | 12.18%  (48) | 37.06%  (146) | 12.94%  (51) |

Table S3. Descriptive statistics of survey 2-5 questions and responses.

| **2-5 Participation and engagement in online peer support group, (%), (n)** | | | | | |
| --- | --- | --- | --- | --- | --- |
| **Items** |  | | | | |
|  | Strongly disagree (1) | Disagree (2) | Neither agree nor disagree (3) | Agree (4) | Strongly agree (5) |
| Item a) | 12.83%  (49) | 13.35% (51) | 28.53%  (109) | 28.80%  (110) | 16.49%  (63) |
| Item b) | 8.12%  (31) | 7.59% (29) | 35.60%  (136) | 22.51%  (86) | 26.18%  (100) |
| Item c) | 4.45%  (17) | 4.71% (18) | 12.30%  (47) | 29.06%  (111) | 49.48%  (189) |

Table S4. Descriptive statistics of survey 2-6 questions and responses.

| **2-6 Willingness to share information, (%), (n)** | | | | | |
| --- | --- | --- | --- | --- | --- |
| **Items** |  | | | | |
|  | Strongly disagree (1) | Disagree (2) | Neither agree nor disagree (3) | Agree (4) | Strongly agree (5) |
| Item a) | 1.83%  (6) | 3.67% (12) | 20.80%  (68) | 40.06%  (131) | 33.64%  (110) |
| Item b) | 5.50%  (18) | 7.65% (25) | 21.10%  (69) | 40.67%  (133) | 25.08%  (82) |
| Item c) | 0.92%  (3) | 5.50% (18) | 15.29%  (50) | 41.90%  (137) | 36.39%  (119) |

Table S5. Descriptive statistics of survey 2-7 questions and responses.

| **2-7 Ease of use, (%), (n)** | | | | | |
| --- | --- | --- | --- | --- | --- |
| **Items** |  | | | | |
|  | Strongly disagree (1) | Disagree (2) | Neither agree nor disagree (3) | Agree (4) | Strongly agree (5) |
| Item a) | 0.60%  (2) | 3.27% (11) | 9.82%  (33) | 44.35%  (149) | 41.96%  (141) |
| Item b) | 0.89%  (3) | 6.25% (21) | 13.69%  (46) | 45.24%  (152) | 33.93%  (114) |
| Item c) | 2.68%  (9) | 7.14% (24) | 26.79%  (90) | 40.48%  (136) | 22.92%  (77) |
| Item d) | 1.19%  (4) | 1.19%  (4) | 13.69%  (46) | 39.99%  (131) | 44.94%  (151) |

Table S6. Descriptive statistics of survey 2-8 questions and responses.

| **2-8 Information utility, (%), (n)** | | | | | |
| --- | --- | --- | --- | --- | --- |
| **Items** |  | | | | |
|  | Strongly disagree (1) | Disagree (2) | Neither agree nor disagree (3) | Agree (4) | Strongly agree (5) |
| Item a) | 0.82%  (3) | 3.02% (11) | 10.71%  (39) | 44.23%  (161) | 41.21%  (150) |
| Item b) | 1.65%  (6) | 6.32% (23) | 18.96%  (69) | 46.98%  (171) | 26.10%  (95) |
| Item c) | 4.76%  (16) | 15.18% (51) | 31.25%  (105) | 36.01%  (121) | 12.80%  (43) |
| Item d) | 6.55%  (22) | 23.81% (80) | 27.38%  (92) | 30.95%  (104) | 11.31%  (38) |

Table S7. Descriptive statistics of survey 2-9 questions and responses.

| **2-9 Privacy and security concerns, (%), (n)** | | | | | |
| --- | --- | --- | --- | --- | --- |
| **Items** |  | | | | |
|  | Strongly disagree (1) | Disagree (2) | Neither agree nor disagree (3) | Agree (4) | Strongly agree (5) |
| Item a) | 16.04%  (51) | 17.92% (57) | 30.19%  (96) | 20.13%  (64) | 15.72%  (50) |
| Item b) | 33.65%  (107) | 20.44% (65) | 19.81%  (63) | 19.81%  (63) | 6.29%  (20) |
| Item c) | 41.51%  (132) | 23.58% (75) | 23.58%  (75) | 8.49%  (27) | 2.83%  (9) |
| Item d) | 5.97%  (19) | 14.15% (45) | 50.94%  (162) | 24.21%  (77) | 4.72%  (15) |
